# Statistical and radiobiological analysis of the so-called thyroid stunning

**DOI:** 10.1186/s13550-015-0144-9

**Published:** 2015-11-19

**Authors:** Stephan Walrand, Michel Hesse, François Jamar

**Affiliations:** Nuclear medicine, Molecular Imaging, Radiotherapy and Oncology Unit (MIRO), IECR, Université Catholique de Louvain, Av. Hippocrate 10, 1200 Brussels, Belgium

**Keywords:** Thyroid stunning, Radiobiological model, TCP, Survival fraction, Radio-iodine

## Abstract

**Background:**

The origin of the reduction in thyroid uptake after a low activity iodine scan, so-called stunning effect, is still controversial. Two explanations prevail: an individual cell stunning that reduces its capability to store iodine without altering its viability, and/or a significant cell-killing fraction that reduces the number of cells in the tissue still taking up iodine. Our aim is to analyze whether this last assumption could explain the observed reduction.

**Methods:**

The survival fraction after administration of a small radioiodine activity was computed by two independent methods: the application of the statistical theory underlying tissue control probability on recent clinical studies of thyroid remnant ^131^I ablation and the use of the radiosensitivities reported in human thyroid cell assays for different radioiodine isotopes.

**Results:**

Both methods provided survival fractions in line with the uptake reduction observed after a low ^131^I activity scan. The second method also predicts a similar behavior after a low ^123^I or ^124^I activity scan.

**Conclusions:**

This study shows that the cell-killing fraction is sufficient to explain the uptake reduction effect for ^131^I and ^123^I after a low activity scan and that even if some still living cells express a stunning effect just after irradiation (as shown in vitro), they will mostly die with time. As the β/α value is very low, this therapy fractionation should not impact the patient outcome in agreement with recent studies. However, in case of huge uptake heterogeneity, pre-therapy scan could specifically kills high-uptake cells and by the way could reduce the cross irradiation to the low-uptake cells during the therapy, resulting in a reduction of the ablation success rate.

**Electronic supplementary material:**

The online version of this article (doi:10.1186/s13550-015-0144-9) contains supplementary material, which is available to authorized users.

## Background

The so-called thyroid stunning effect refers to the observed fact that the ^131^I uptake at therapy is lower than the one predicted from a ^131^I pre-therapeutic scan, even when using a low activity such as 74 MBq [1–7]. In addition to a reduction of the iodine uptake at 24 h, a reduction of the effective half-life was also reported [7]. The explanation for this uptake reduction is still controversial [8–14]. In vitro studies suggest that irradiation may perturb cell metabolism before cell viability [15], specifically by a downregulation of the sodium/iodide symporter (NIS) expression at the transcription level [16, 17], resulting in a reduction of the capacity to transport iodine. Others suggest that this reduction could purely result from killed cells that are thus no longer present for (or able of) keeping accumulated iodine [9]. The first hypothesis really corresponds to a cell stunning, while the latter one corresponds to a therapy fractionation.

A lower, but still actual uptake reduction was also observed by Hilditch et al. [6] after administering 200 MBq of ^123^I. To explain this fact, the authors also suggested a self-stunning effect occurring during the ^131^I therapy itself. The rationale is that in average ^123^I delivers 80 times less Gy per GBq than ^131^I due to its shorter half-life and its lower electron emission energy [18]. However, Lundh et al. [19] showed in thyroid cells assays that the relative biological effectiveness (RBE) of ^123^I was about 5-fold higher than that of ^131^I, and thus an uptake reduction induced by a low ^123^I activity cannot a priori be rejected.

The aim of this study is to determine whether the cell-killing fraction can quantitatively explain the uptake reductions of thyroid remnants reported after a low activity pre-therapeutic scan using either radio-iodines (^131^I, ^123^I) [6, 7]. The survival fraction, or equivalently the cell-killing fraction, was computed by two independent approaches: analyzing data reported in recent thyroid ablation studies [20–24] with the statistical theory underlying the tissue control probability (TCP) [25], and using the radiosensitivities measured in human thyroid cell assays performed on both normal and malignant lines [26] and the mean absorbed doses reported in clinical studies [7]. In addition, the second computation is also presented in Additional file 1 for a heterogeneous absorbed dose distribution as observed on ^124^I PET scans [27].

## Methods

### Pure statistical analysis

Simply assuming Poisson statistics, Brahme and Agren [25] deduced for TCP (initially named eradication probability), a relation that is known widely accepted [28, 29]:

1 $$ \mathrm{T}\mathrm{C}\mathrm{P}(A) = {e}^{-\mathrm{N}\mathrm{c}\ \mathrm{S}\mathrm{f}\left(D(A)\right)} $$

where *A* is the administered activity, D(A) is the absorbed dose in the tissue, Sf is the cell surviving fraction, and Nc is the number of cells alive before irradiation.

As beta-particles present a low linear energy transfer (LET) and as the effective half-life of ^131^I in thyroid is sufficiently large to result in low dose rate, the survival fraction Sf can be approximated by:

2 $$ \mathrm{S}\mathrm{f}\left(D(A)\right) = {e}^{-\alpha D(A)} $$

where *α* is the cell radiosensitivity.

Therapeutics ^131^I activities still correspond to chemical traces (less than 0.5 μg), thus for each patient, the absorbed dose is proportional to the administered activity, and Sf can be rewritten as:

3 $$ \mathrm{S}\mathrm{f}\left(D(A)\right) = {e}^{-\widehat{\alpha}\ A} $$

In addition to the cells radiosensitivity *α*, the parameter $\widehat{\alpha}$ also takes into account the uptake and the *S* factor of the thyroid remnant.

Recent studies [20–24] using a single administration of 1.11 GBq of ^131^I report thyroid remnant control probabilities ranging from 70 to 95 %, i.e.,

4 $$ \mathrm{T}\mathrm{C}\mathrm{P}\left(1.11\;\mathrm{GBq}\right)\in \left[0.70,\ 0.95\right] $$

### Thyroid cells radiosensitivity

Gaussen et al. [26] measured thyroid normal and tumor cells radiosensitivities for low LET from ^60^Co irradiation of 15 cell cultures (5 normal, 6 papillary carcinoma, 1 follicular carcinoma, 3 macrofollicular adenoma, coming from 11 patients). These assays analyzed according to the linear-quadratic model showed that *α* ranged from 0.37 Gy^−1^ (most radioresistant tumor cells) up to 0.97 Gy^−1^ (most radiosensitive normal cells), with a very low β/α value. As beta particles also present a low LET, these *α* values measured for ^60^Co can be used for ^131^I as recommended by ICRP [30]. Furthermore, the quadratic term can be neglected, validating the use of Eq. (2). Lundh et al. [19] showed in thyroid cells assays that the relative biological effectiveness (RBE) of ^123^I versus ^131^I was about 5-fold, resulting in *α* value for ^123^I ranging from 1.9 to 4.9 Gy^−1^. This can be explained by the larger LET of ^123^I Auger electrons as compared to that of higher energy ^131^I beta particles. In addition, a higher LET also induces an even lower β/α value [31].

### Cell survival fraction in thyroid remnants

As thyroid remnants are an unknown mix of normal and tumor cells, the cell survival fraction was estimated within the measured range of *α* values.

Lassman et al. [7] measured the uptake in the thyroid remnants of six patients in two consecutive scans after administration of 74 MBq of ^131^I (delay 4–6 weeks). They found that the absorbed dose at the first scan ranged from 4 to 38 Gy, corresponding to 0.15 and 1.70 % of the administered activity per gram of tissue, together with a biological half-life larger than 40 days. Rescaled to the standard thyroid mass (20 g), this gives an uptake ranging from 3 to 34 %. This is in line with iodide biokinetics reported from ICRP model giving an uptake of 33 % for a normal thyroid and a biological half-life of 80 days [32].

Hilditch et al. [6] reported the therapeutic to diagnostic uptake ratio of thyroid remnants in 26 and 16 patients using 120 MBq of ^131^I and 200 MBq of ^123^I for the diagnostic scan, respectively. As the thyroid remnant uptakes were not reported, we estimated the cell survival fraction by taking also into account the variability of uptake in thyroid remnants derived in the previous paragraph from [7].

As the biological half-life (>40 days) is much larger than the ^124^I and ^123^I physical half lives, i.e., 4.18 and 0.55 days, these latter values were used as the effective half-lives in the absorbed dose computation.

## Results

### Pure statistical analysis

Straight inversion of Eq. 1 gives

5 $$ \mathrm{S}\mathrm{f}(A) = \frac{- \ln \left(\mathrm{T}\mathrm{C}\mathrm{P}(A)\right)}{\mathrm{Nc}} $$

For 0.02 to 2 g of thyroid remnant, i.e., Nc ∈ [10^7^, 10^9^] [33], Eqs. 4, 5 give

6 $$ \mathrm{S}\mathrm{f}\left(1.11\kern0.5em \mathrm{GBq}\right)\in \left[5.1\times {10}^{-10},3.6\times {10}^{-8}\right] $$

Note that, due to the random nature of energy deposition in cells by the beta particles, a value Nc × Sf significantly lower than one is needed to get more than 70 % of chance to kill all the cells.

Combining Eqs. 3 and 6, we can write

7 $$ \mathrm{S}\mathrm{f}\left(74\;\mathrm{MBq}\right)={e}^{-\overline{\alpha}\ 74\;\mathrm{MBq}} = {\left({e}^{-\overline{\alpha}\ 1.11\mathrm{GBq}}\right)}^{74/1110} = {\left(\mathrm{S}\mathrm{f}\left(1.11\kern0.5em \mathrm{GBq}\right)\right)}^{1/15}\in \left[24\%,32\%\right] $$

As a result of the 15th root, the survival fraction for 74 MBq only slightly varies in the range of TCP values reported after administration of 1.11 GBq of ^131^I.

The same computation for 120 MBq of ^131^I gives

8 $$ \mathrm{S}\mathrm{f}\left(120\kern0.5em \mathrm{MBq}\right)\in \left[10\%,16\%\right] $$

**Fig. 1:**
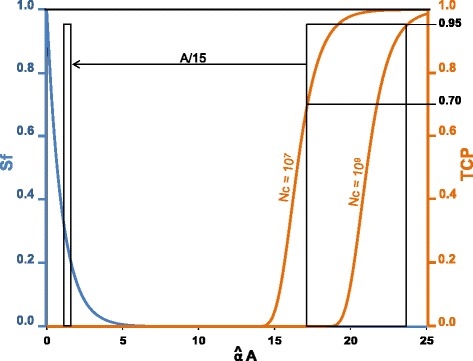
Predicted survival fraction (Eq. 3) and TCP (Eq. 1) for 10^7^ and 10^9^ cells as functions of the dimensionless variable $\widehat{\alpha}A$. Note that an activity 15 times lower than that needed to ensure a TCP between 70 and 95 % already kills about 75 % of the cells. The figure depends only on the cells number ,Nc, present in the tissue to be controlled and not on their type. The cell type only modifies the value of $\widehat{\alpha}$, i.e., the corresponding activity *A* needed to control the tissue. 10^7^ and 10^9^ cells typically correspond to thyroid remnants total weight ranging from 0.02 to 2 g

Figure 1 shows the predicted survival fraction Sf and the TCP for 10^7^ and 10^9^ cells as functions of the dimensionless variable $\widehat{\alpha}A$. Note that this figure depends only on the number of cells ,Nc, in the tissue to be controlled and not on the cell type. This last feature only modifies the value of $\widehat{\alpha}$, or in the other words, the corresponding activity *A* needed to control the tissue.

**Fig. 2:**
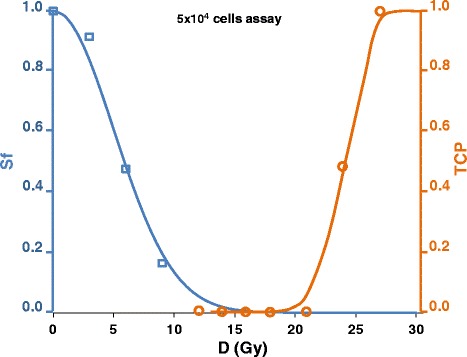
Cell survival fraction Sf and TCP measured by Kappler et al. after uniform irradiation of sarcoma megacolony assays (5 × 10^4^ cells) (curves Lu-siRNA in Figs. 5 and 6 of [31]: cells were radio-sensitized by RNA transfection). The much larger number of cells in actual lesions (>10^7^→Sf < 10^−8^ for TCP = 0.9) still dramatically widens the width of the valley in between the two curves as shown in Fig. 1

### Radiobiological analysis

Table 1 shows the mean absorbed dose and the cell survival fraction computed assuming uniform absorbed dose for the pre-therapeutic activities administered in Sgouros et al. [27], Lassmann et al. [7], and Hilditch et al. [6]. The mean absorbed dose $\overline{D}$ was computed using Olinda [18]. Lowest and highest mean absorbed dose values correspond to the minimal and maximal thyroid remnants uptake observed in [7], i.e., 0.15 and 1.7 %/g, respectively. Lowest and highest (italicized in Table 1) cell survival fractions were derived from the combination of the maximal and minimal (italicized in Table 1) absorbed dose and *α* values, respectively.

**Table 1:** Summary of reported and computed data

		Uptake min–max	Cells tum.–nor.		Uptake ratio	Dose dist. uniform
	*A* (MBq)	$\overline{D}$ (Gy)	α (Gy^−1^)	$\alpha \overline{D}$		*S*_*f*_ Eq. 2
	*A* (MBq)	$\overline{D}$ (Gy)	α (Gy^−1^)	$\alpha \overline{D}$		*S*_*f*_ Eq. 2
^124^I [26]	74	*2.1*–23.7	*0.37*–0.97	*0.78*–23.0		0–*0.46*
^131^I [7]	74	*4.0*–38.0	*0.37*–0.97	*1.48*–36.9	0.08–1.00	0–*0.23*
^131^I [6]	120	*6.5*–61.6	*0.37*–0.97	*2.41*–59.8	0.05–0.83	0–*0.09*
^123^I [6]	200	*0.1*–1.3	*1.85*–4.85	*0.19*–6.3	0.17–0.86	0–*0.83*

## Discussion

Two independent formalisms show that the cell-killing fraction is sufficient to explain the observed iodine uptake reduction after a first pre-therapy scan, i.e., (1) a statistical analysis based on Poisson statistics and on the remnant control probability reported in recent clinical studies, and (2) a radiobiological analysis using normal and cancer thyroid cells radiosensitivity assays data jointly with the uptake non-uniformity observed in ^124^I PET.

Note that such explanation of the uptake reduction by cells killing is not incompatible with studies reporting a decrease of the uptake reduction when the delay between the two scans increases [34, 35]. Indeed, after irradiation, a fraction of the surviving cells will proliferate with time and by the way the iodine uptake will also re-increase with time.

The pure statistical analysis shows that, contrary to a commonly accepted opinion, small activities of ^131^I such as 74 or 120 MBq, i.e., one order of magnitude smaller than those used for therapy, already kill in average about 75 or 87 % of the remnant cells, respectively. This is sufficient to explain the mean uptake reduction, i.e., 56 % for 74 MBq and 67 % for 120 MBq, observed by Lassman et al. [7] and by Hilditch et al. [6], respectively.

Equation 1 involves that a probability of 70 % of ablating a 1 g of tissue requires a survival fraction lower than 10^−9^. This corresponds to absorbed doses far above those that already kill a significant fraction of cells as shown in Fig. 1. The pattern of Fig. 1, i.e., a large valley between the Sf and the TCP curves, was also experimentally observed in direct measurements of the survival fraction and of the TCP by Kappler et al. [36] in megacolony assays after uniform irradiation (Fig. 2). This general pattern is not related to the cell type, but is related to the random energy deposition of rays jointly with the high number of cells in tissues that involves large absorbed dose in order to kill almost all the cells.

The radiobiological computation using Eq. 2 and the radiosensitivities measured in thyroid cell assays confirmed the result of the statistical analysis, i.e., that the therapy to diagnostic uptake ratios can be explained by the cell-killing fraction (Table 1). The computed uptake reductions are even too large.

Lassman et al. reported mean absorbed doses ranging from 4 to 38 Gy (Table 3 in [7]) for a first 74 MBq ^131^I scan. Rescaled to a 1.11 GBq ^131^I therapy without pre-therapy scan, the mean absorbed dose typically ranges from 60 to 600 Gy. Assuming uniform absorbed dose, such huge values should provide a TCP equal to 100 %, higher than the 70–95 % range reported in the literature [20–24]. Taking into account the heterogeneous uptake, as measured in ^124^I PET [27] and using a more sophisticated radiobiological model, the computed survival fraction increased and provided uptake reduction in line with the observations (see Additional file 1).

It is a real deficiency that in all the papers reporting the stunning effect in vivo [1–6], except that of Lassmann et al. [7], neither the absorbed doses nor the thyroid remnant uptakes were assessed. Regarding the huge variation in remnant uptakes due to the lesion type, but also due to the hypo- or eu-thyroid status of the patient, doses estimation is required to avoid blurring of the observations that could lead to misunderstanding of the physical effects.

The present study shows that the cell-killing fraction is sufficient to explain the observed reductions in uptake, and that even if some living cells express a stunning effect just after irradiation (as shown in vitro [15–17]), they will mostly die with time. In other words, the pre-therapy scan appears to be itself a first therapy cycle. As the β/α value is very low (0.02 ± 0.03 Gy^−1^ [27]), no significant reduction of the therapeutic efficiency should result from this absorbed dose fractionation. This is in line with several recent studies reporting that the uptake reduction does not impact the ^131^I therapy outcome [4, 37–40].

However, one has to keep in mind that in lesions expressing huge uptake heterogeneity, the low pre-therapy activity could kill cells with high uptakes without killing by cross firing surrounding cells with low uptakes. Afterwards, these cells with high uptake will no longer take ^131^I up during the main therapy session (because most are killed), reducing by this way the cross irradiation that the cells with low uptake could have received. This cell type selection occurring during the pre-therapy scan could explain lower rates of ablation success reported in some studies [41, 42].

## Conclusions

The pure statistical analysis supports that the so-called thyroid stunning effect can be explained by the cell-killing fraction. This result is confirmed by the quantitative radiobiological analysis based on normal and tumor cells radiosensitivities measured on human thyroid cell assays.

The study also shows that the two radioiodine isotopes (^131^I, ^123^I) induce an uptake reduction after a low activity scan and that it could be the case for ^124^I too. As the β/α value is very low, this effective fractionation of the therapy should not impact the patient outcome, in agreement with recent studies. However in case of huge uptake heterogeneity, pre-therapy scan could specifically kills high-uptake cells and by the way could reduce the cross irradiation to the low uptake cells during the therapy, resulting in a reduction of the ablation success rate.

## Additional file

Additional file 1:
**Prediction of the uptake reduction after a first low activity** ^**131**^ **I or** ^**123**^ **I scan observed by Hilditch et al. [** 6 **] and Lassmann et al. [** 7 **] using the thyroid cells radiosensitivity [** 26 **] jointly with the non-uniform uptake in tissue observed in** ^**124**^ **I PET [** 27 **]**. (DOCX 102 kb)
