# Supplementary material for: Statistical and radiobiological analysis of the so-called thyroid stunning
Source: EJNMMI Res. 2015 Nov 19;5:67. doi: 10.1186/s13550-015-0144-9 (PMC4651970; doi:10.1186/s13550-015-0144-9)
Supplement: Additional file 1: — Prediction of the uptake reduction after a first low activity 131 I or 123 I scan observed by Hilditch et al. [ 6 ] and Lassmann et al. [ 7 ] using the thyroid cells radiosensitivity [ 26 ] jointly with the non-uniform uptake in tissue observed in 124 I PET [ 27 ]. (DOCX 102 kb) [file 13550_2015_144_MOESM1_ESM.docx]

**Statistical and radiobiological analysis of the so-called thyroid stunning.**

Running foot line: Radiobiological analysis of the thyroid stunning effect.

Stephan Walrand, Michel Hesse, François Jamar.

Nuclear medicine, Molecular Imaging, Radiotherapy and Oncology Unit (MIRO), IECR, Université Catholique de Louvain, Brussels, Belgium

Corresponding author:

Stephan Walrand

Nuclear medicine

Av. Hippocrate 10

1200 Bruxelles

Belgium

tel: 32 2 7642592

fax: 32 2 7645408

[Stephan.walrand@uclouvain.be](mailto:Stephan.walrand@uclouvain.be)

**Appendix A**

*Radiobiological model*

The end point cell survival fraction $S^{\infty}$ after a heterogeneous delivered dose is (β ≈ 0):

$S^{\infty}= \int_{0}^{\infty} f\left( D \right) e^{-\alpha D} dD$ (1A)

where *f(D)* is the normalized frequency distribution of the absorbed dose inside the lesions. When the absorbed dose is uniform we get:

$f_{u}\left( D \right)= \delta(D-\bar{D} )$ (2A)

where δ is the Dirac distribution and $\bar{D}$ is the mean dose. In this case the survival fraction reduces to the well known relation:

$S_{u}^{\infty}= e^{-\alpha\overline{D}}$. (3A)

For non-uniform dose distribution, *f(D)* is related to the dose volume histogram *DVH(D)* by:

$f\left( D \right)= -\frac{dDVH(D)}{dD}$ (4A)

As $DVH(0)=1$ and $DVH\left( \infty\right)=0$ we naturally have the normalization of *f(D)*:

$\int_{0}^{\infty} f\left( D \right)dD=1$ (5A)

Sgouros et al. [27] reported DVH of nine thyroid metastases in two patients assessed by ^124^I PET imaging: one patient with 4 big necrotic lesions that are thus not representative of thyroid remnants, and the second one with 5 small lesions. We modeled the average of the DVH of these last 5 lesions by the function:

$DVH\left( D \right)= {c e}^{-\frac{a\left( D-\varepsilon\overline{D} \right)}{\overline{D}}}+{\left( 1-c \right)e}^{-\frac{b\left( D-\varepsilon\overline{D} \right)}{\overline{D}}} if D>\varepsilon\bar{D}$

$=1 otherwise$ (6A)

where the small dose $\varepsilon\bar{D}$ is the absorbed dose due to cross irradiation from neighbor cells: cells with very low uptake will at least receive a small irradiation proportional to the mean dose. The three dimensionless parameters *a,b* and *ε* were fitted to the measured DVH [27] with c constrained to ensure the following identity:

$\bar{D}=\int_{0}^{\infty} f\left( D \right) D dD= \left( c\left( \varepsilon+\frac{1}{a} \right)+(1-c)\left( \varepsilon+\frac{1}{b} \right) \right)\bar{D}$ (7A)

Or more explicitely:

$c= \left( \varepsilon+\frac{1}{b} \right)/\left( \frac{1}{b}-\frac{1}{a} \right)$ (8A)

The second equality of eq.7A is obtained from an integration by parts.

A direct integration of eq. 1A together with eq. 4A and 6A gives:

$S^{\infty}= \left( \frac{c}{1+\alpha\overline{D}/a}+\frac{1-c}{1+\alpha\overline{D}/b} \right) e^{-\varepsilon\alpha\overline{D}}$ (9A)

The dose is delivered in several days, and cell death occurs after a variable delay. Thus the living cells number with time can be written as:

$N\left( t \right)= S^{\infty} N_{c}+ \left( 1-S^{\infty} \right) N_{c} e^{-\lambda t}$ (10A)

Where $S^{\infty}$ is the final survival fraction and Nc is the number of clonogenic cells before irradiation. The first term of left hand side of eq. 9A is the number of cells preserved form lethal damages, and the second term the number of cells lethally targeted and dying with an half life ln(2)/λ. In eq. 9A it was assumed that the delay between the two scans is too short to allow a significant cells repopulation.

The time dependent cell survival fraction is thus:

$S\left( t \right)= S^{\infty}+\left( 1-S^{\infty} \right)e^{-\lambda t}$ (11A)

where λ is related to the dying cell half life.

The tumor control probability (TCP) is given by [25]:

$TCP= e^{-N_{c} S^{\infty}}$ (12A)

where N_c_ is the number of clonogenic cells before irradiation.

**Results:**

Figure 1A shows the DVH averaged on the 5 tumors individually assessed by Sgouros et al. from ^124^I PET imaging (figure 7 in [27]), together with its fit based on eq. 6A.

Figure 2A shows the reduction of the uptake as a function of the delivered dose to thyroid remnants from the first diagnostic scan as assessed by Lassmann et al. [7] compared to the survival fraction computed with eq. 8A for the non-uniform dose distribution.

Figure 3A shows the comparison of the uptake reduction measured by Hilditch et al. [6] with the time dependent survival fraction (eq. 10A) computed for the non-uniform dose distribution within the measured range of thyroid remnants uptakes and of thyroid cells α values. The best fit was obtained with λ = 0.16 day^-1^. Note that the λ value impacts the curve only during the first 20 days.

**Discussion**

The radiobiological computation shows that the therapy to diagnostic uptake ratios measured by Hilditch et al. [6] for 120 MBq of ^131^I or 200 MBq of ^123^I diagnostic scans can be explained simply by the cell killing fraction (figure 3A). The reductions in uptake are quite spread as a result of the variable doses received by the thyroid remnants. Unfortunately neither these doses, nor the thyroid remnant uptakes were reported in [6].

The comparison shows that the reduction of radioiodine uptake measured by Lassmann et al. [7] in 3 patients (purple squares in Fig. 1) is much lower than the predicted cell survival fraction. These 3 patients displayed at the first diagnostic day a much lower thyroglobulin level (< 1.8 ng/mL) than the 3 other ones (> 3.5 ng/mL), although the link between this observation and the uptake reduction discrepancy is not clear. Indeed, as the data are expressed as functions of the measured absorbed dose, difference in uptake or mass between the two thyroglobulin populations are already taken into account. As there was no validated method to estimate the remnant mass, Lassmann et al. used the remnant area (measured on planar view) times a mean thickness of 2 mm. This previously published method was based on surgeon's operative notes and from discussions with the surgeon [43,44]. This method suffers from three issues: the remnant thickness is clearly dependent on the surgerical act, a remnant is not always imaged along the direction perpendicular to its surface and after surgery the empty thyroid cavity collapses, such as a removed glove, sticking together different remnants (see Fig. 3 in [7] showing all the remnants activity located along a central line). These two last effects induce a larger effective remnant thickness which could result in an overestimation of the doses as assessed in [7] using a standard thickness of 2 mm.

The use of a simple bi-exponentially decreasing model for the dose distribution allows easy radiobiological computations that can be reproduced by the reader. Regarding the variability of the dose distribution observed in thyroid metastases [21,45], choosing more sophisticated dose distribution models does not really make sense. Improvement of the quantitative radiobiological analysis of the uptake reduction should require the actual assessment of the dose distribution of each lesion.

The present study also shows the significant impact of the dose heterogeneity on the 50% cell survival fraction region (table 1A). The impact on the 10^-8^ cell survival fraction region is still much huger, as a very small volume not well irradiated is sufficient to preserve more than one cell out of a hundred millions. This dramatically increases the doses needed to cure metastases and ablate thyroid remnants. The present model shows that for thyroid remnants having an uptake equal to the mean value 0.66 %/g observed in [7], an administered activity ^131^I of 2.5 GBq (≈70 mCi) is sufficient for the ablation. However, one has to keep in mind that the thyroid remnant uptake per gram is highly variable, 0.15 to 1.70 %/g as assessed in [7]. As a result the administered activity to control a thyroid remnant ranges from 0.6 to 6.5 GBq depending on its uptake. This clearly supports the benefit to perform a pre-therapeutic dosimetry in order to administer an activity sufficient for patients having thyroid remnants with low uptake and also to avoid unjustified organ exposures for patients having remnants with high uptake. The added value of this should however be evaluated prospectively, in particular when the trend in thyroid carcinoma ablation is to reduce drastically activities from an historical standard of '100 mCi' to an emerging standard of '30 mCi' [46]. Besides this, the study shows that pretherapeutic dosimetry in patients with metastases, which is advocated by many authors, does not preclude full efficiency of the therapy.

For the time being, the present model used parameters (*a* and *ε*) derived from a limited number of lesions (5 from [27]) and cannot thus strictly be used as such in individualized thyroid ablation planning. The major merit of the model is to provide a unified quantitative comprehension of the so-called thyroid stunning effect and of the TCP both observed in clinical therapy.

**References**

43) Maxon HR III, Englaro EE, Thomas SR, et al. Radioiodine-131 therapy for well-differentiated thyroid cancer: a quantitative radiation dosimetric approach—outcome and validation in 85 patients. J Nucl Med. 1992;33:1132–1136.

44) Thomas SR, Maxon HR, Kereiakes JG, Saenger EL. Quantitative external counting techniques enabling improved diagnostic and therapeutic decisions in patients with well-differentiated thyroid cancer. Radiology. 1977;122:731–737.

45) Sgouros G, Hobbs RF, Atkins FB, Van Nostrand D, Ladenson PW, Wahl RL. Three-dimensional radiobiological dosimetry (3D-RD) with 124I PET for 131I therapy of thyroid cancer. Eur J Nucl Med Mol Imaging. 2011;38:S41-7.

46) Schlumberger M, Catargi B, Borget I, Deandreis D, Zerdoud S, Bridji B, Bardet S, Leenhardt L, Bastie D, Schvartz C, Vera P, Morel O, Benisvy D, Bournaud C, Bonichon F, Dejax C, Toubert ME, Leboulleux S, Ricard M, Benhamou E. Strategies of radioiodine ablation in patients with low-risk thyroid cancer. N Engl J Med. 2012;366:1663-73.

**Figures**

Figure 1A: fit with eq. 6A (brown curve) of the DVH averaged (blue curve) on the 5 tumors individually assessed by Sgouros et al. from ^124^I PET imaging (figure 7 in [27]).

Figure 2A: Squares: uptake reduction factor as a function of the mean dose $\bar{D}$ measured by Lassmann et al. in six patients (table 3 in [7]). Purple and blue squares correspond to 2 sets of 3 patients having at the first diagnostic scan a thyroglobulin level lower than 1.8 ng/mL and higher than 3.5 ng/mL, respectively. Blue area: cell survival fraction predicted using eq. 8A within the measured α ranges, i.e. from normal to tumor cells as shown by arrows.

Figure 3A: Squares and bullets: therapy to diagnostic uptake ratio measured by Hilditch et al. (i.e. extracted from figure 1 in [6]). Brown and blue area: time-dependent cell survival fraction computed with eq. 9A for the non-uniform dose distribution (eq. 6A) within the measured range of thyroid remnant's uptakes and of cell α values (see table 1).

**Table**

Table 1: summary of reported and computed data.

|  |  | uptake  min-max | cells  tum.-nor. |  | dose dist.  uniform | dose dist.  non-uniform |
| --- | --- | --- | --- | --- | --- | --- |
|  | A  (MBq) | $\bar{D}$  (Gy) | α  (Gy^-1^) | $\alpha\bar{D}$ | $S_{u}^{\infty}$ eq. 3A  (%) | $S^{\infty}$ eq. 8A  (%) |
| ^124^I [27] | 74 | **2.1**-23.7 | **0.37**-0.97 | **0.78**-23.0 | 0-**46** | 2-**61** |
| ^131^I [7] | 74 | **4.0**-38.0 | **0.37**-0.97 | **1.48**-36.9 | 0-**23** | 0-**48** |
| ^131^I [6] | 120 | **6.5**-61.6 | **0.37**-0.97 | **2.41**-59.8 | 0- **9** | 0-**37** |
| ^123^I [6] | 200 | **0.1**- 1.3 | **1.85**-4.85 | **0.19**- 6.3 | 0-**83** | 17-**85** |
